# Measuring Neglect

**DOI:** 10.1371/journal.pntd.0000118

**Published:** 2007-11-28

**Authors:** Peter Hotez

**Affiliations:** Sabin Vaccine Institute and Department of Microbiology, Immunology, and Tropical Medicine, George Washington University Medical Center, Washington, D. C., United States of America

As a basis for prioritizing the allocation of financial and other resources targeted for international development, the strong preference of the global community's policy makers and donors is to rely on numbers. Metrics such as DALYS (disability-adjusted life years—the number of healthy life years lost either from death or disability) or DALYs averted per dollar provide highly useful information about the cost-effectiveness of health interventions in different settings. In turn, the cost-effectiveness of a program is considered fundamental to its merit or its need for redesign. In this regard, one of the important achievements of this decade is the publication of the Second Edition of the *Disease Control Priorities in Developing Countries. DCP-2* is a landmark document for informing fundamental policy considerations, selecting and scaling up effective interventions, and identifying opportunities for ongoing research [1].

Among the important findings of the *DCP-2* is the great cost-effectiveness of many interventions that target the neglected tropical diseases (NTDs). For example, at a cost of US$2–US$9 per DALY averted for deworming (mass drug administration for soil-transmitted helminth infections) and US$4–US$9 per DALY averted for yearly antifilarial preventive chemotherapy treatments, these two NTD control programs are now considered some of the better buys in public health, compared to, for example, US$257–US$4,565 per DALY averted for measles vaccination (which is still a very good buy) [2]. Global efforts to integrate NTD control by combining interventions in a package of antihelminthic drugs together with azithromycin is expected to result in additional cost savings of 26%–47% [3], making NTD control an even better buy [4].

In some respects, however, we have only started to explore the true disease burden of the NTDs and therefore the ultimate cost-effectiveness of NTD interventions. For example, Professor Charles H. King and his colleagues have begun to incorporate a number of additional chronic and debilitating elements into their estimates of the disease burden of schistosomiasis and have determined that the number of DALYs lost from this disease may far exceed previous estimates [5],[6]. Over the next two years, an initiative sponsored by the new Institute of Health Metrics and Evaluation at the University of Washington in Seattle, together with the Department of International Health at the Johns Hopkins Bloomberg School of Public Health and the Global Network for Neglected Tropical Diseases, will revisit the disease burden for most of the NTDs [7]–[9].

In anticipation of renewed interest in assessing the disease burden of the NTDs and the cost-effectiveness of NTD interventions, *PLoS Neglected Tropical Diseases* will devote a considerable amount of space and energy to these issues. Under the leadership of Simon Brooker and Juerg Utzinger, in this issue of *PLoS Neglected Tropical Diseases* we begin a series of articles devoted to NTD disease burden and the controversies about how the chronic and disabling features of these conditions should be best evaluated. It is hoped that this dialogue will both help to stimulate new interest in the NTDs and elevate their profile among global health policy makers. The series begins with an overview by Colin Mathers and colleagues of the analytic approach used by the original Global Burden of Disease (GBD) Study, with a particular focus on the NTDs [10]. The overview is accompanied by a provocative Viewpoint from Burton Singer and Carol Ryff, who call for “far more extensive revision of outcome measures and of the entire GBD framework” [11].

Unfortunately, it is not always possible to assign a number to the horrific effects of a NTD. In recognition that there is an almost intangible element that defies conventional quantitative metrics, in this issue of *PLoS Neglected Tropical Diseases* [13], Myrtle Perera of the Marga Institute in Sri Lanka, together with her colleagues at the Universities of Liverpool and Ruhuna, reports on an innovative effort that uses narrative and other qualitative techniques in order to profile the plight of Sri Lankan people living with the effects of lymphatic filariasis (LF). In sometimes graphic detail, Perera et al. eloquently portray the emotional distress and social isolation that results from the stigma of LF, sometimes to the point where affected patients even avoid desperately needed services that are freely available at government clinics. Perera et al. also articulate how these social elements combine in a toxic manner that pushes LF patients into a vortex of poverty, which is almost impossible to escape. Their findings provide an important qualitative complement to the previous financial estimates of Dr. K. D. Ramaiah and his colleagues, who have determined that LF causes more than US$1 billion in economic losses for India annually [14].

The Marga Institute derives its name from a Sanskrit word that translates into a quest for ultimate meaning or deliverance from suffering [15]. The study conducted by the Marga group is certainly in that spirit. By highlighting how the NTDs promote poverty, both quantitatively and through narrative, we at *PLoS Neglected Tropical Diseases* ultimately also aspire to provide a full and complete picture of the sufferings of the world's most vulnerable at-risk populations.

## References

[1] Jamison DT, Breman JG, Measham AR, Alleyne G, Claeson M (2006). Foreword: Disease control priorities in developing countries. 2nd Edition.

[2] Laxminarayan R, Mills AJ, Breman JG, Measham AR, Alleyne G (2006). Advancement of global health: Key messages from the Disease Control Priorities Project.. Lancet.

[3] Brady MA, Hooper PJ, Ottesen EA (2006). Projected benefits from integrating NTD programs in sub-Saharan Africa.. Trends Parasitol.

[4] Hotez PJ, Molyneux DH, Fenwick A, Kumaresan J, Ehrlich Sachs S (2007). Control of neglected tropical diseases.. New Engl J Med.

[5] King CH (2007). Lifting the burden of schistosomiasis—Defining elements of infection-associated disease and the benefits of antiparasite treatment.. J Infect Dis.

[6] King CH, Dickman K, Tisch DJ (2005). Reassessment of the cost of chronic helmintic infection: A meta-analysis of disability-related outcomes in endemic schistosomiasis.. Lancet.

[7] Institute for Health Metrics and Evaluation (2007). Overview.. http://www.healthmetricsandevaluation.org/.

[8] Institute for Health Metrics and Evaluation (2007). Global Burden of Disease Project.. http://www.globalburden.org/.

[9] Global Network for Neglected Tropical Diseases (2007). About the Network.. http://gnntdc.sabin.org/network/index.html.

[10] Mathers CD, Ezzati M, Lopez AD (2007). Measuring the burden of neglected tropical diseases: The Global Burden of Disease Framework.. PLoS Negl Trop Dis.

[11] Singer BH, Ryff CD (2007). Neglected tropical diseases, neglected data sources, and neglected issues.. PLoS Negl Trop Dis.

[12] Humphreys M (2001). Malaria, poverty, race, and public health in the United States.

[13] Perera M, Whitehead M, Molyneux D, Weerasooriya M, Gunatilleke G (2007). Neglected patients with a neglected disease? A qualitative study of lymphatic filariasis.. PLoS Negl Trop Dis.

[14] Ramaiah KD, Das PK, Michael E, Guyatt H (2000). The economic burden of lymphatic filariasis in India.. Parasitol Today.

[15] Marga Institute (2007). http://www.margasrilanka.org/.

